# Odontogenic myxoma of the maxilla: A report of a rare case and review of the literature

**DOI:** 10.4103/0973-029X.64305

**Published:** 2010

**Authors:** Sasidhar Singaraju, Sangeetha P Wanjari, Rajkumar N Parwani

**Affiliations:** *Department of Oral Pathology, Modern Dental College and Research Centre, Airport Road, Gandhi Nagar, Indore - 453 112, India*

**Keywords:** Odontogenic myxomas, fibromyxoma, odontogenic tumors

## Abstract

Odontogenic myxoma represents an uncommon benign neoplasm comprising of 3–6% of all odontogenic tumors. This article presents a rare case of odontogenic myxoma occurring in the maxilla of a 7-year-old male patient with a brief review of the pathogenesis, clinical, radiological, histopathological, ultrastructural and immunohistochemical characteristics of odontogenic myxoma.

## INTRODUCTION

Odontogenic myxoma of the jaws is a rare benign tumor characterized grossly by mucoid or gelatinous grayish-white tissue that replaces the cancellous bone and expands the cortex.[1] It is thought to be of mesenchymal or ectomesenchymal origin.[2] Odontogenic myxomas are locally invasive, nonmetastasizing neoplasms of the jaws, almost exclusively seen in tooth-bearing areas.[2,3]

Most frequently, odontogenic myxomas occur in the second or third decades of life.[2,4,5]

Children and persons over 50 years of age are seldom affected.[2] Myxomas can occur anywhere in the jaws but have a predilection for the molar and premolar regions of the mandible and maxilla. Cortical expansion and perforation are common findings; however, maxillary myxomas often extend into the sinus.[5]

In this article, we present a case of odontogenic myxoma occurring in the maxilla of a 7-year-old boy followed by a review of the literature.

## CASE REPORT

**Figure 1:**
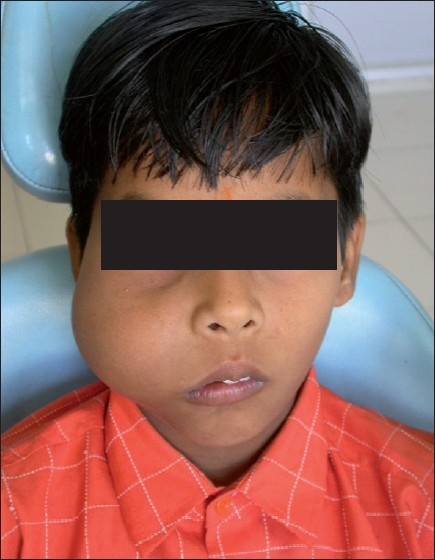
Painless bony hard swelling on the right maxilla, resulting in upward pushing of the right eyeball and eyelid

A 7-year-old boy was referred to Modern Dental College and Research Center, Indore, for the evaluation of a swelling on the right side of his face. His medical history revealed that he developed a fever 18 months prior to his admission, which resolved within 6–7 days, but subsequently developed a mild swelling on the right side involving the middle third of the face. The swelling was painless, but the patient noticed intermittent watery nasal discharge from the right nostril. One and a half years later, when the patient reported to our department, he had a bony hard, nontender swelling of approximately 4 × 3 inches, extending superoinferiorly from the infraorbital ridge to 1 inch above the inferior border of the mandible and anteroposteriorly from the right corner of the mouth to 1.5 cm anterior to the tragus. The borders of the swelling were diffused and the skin overlying the swelling was normal in color. The right lower eyelid and the eyeball were pushed upward [Figure 1].

**Figure 2:**
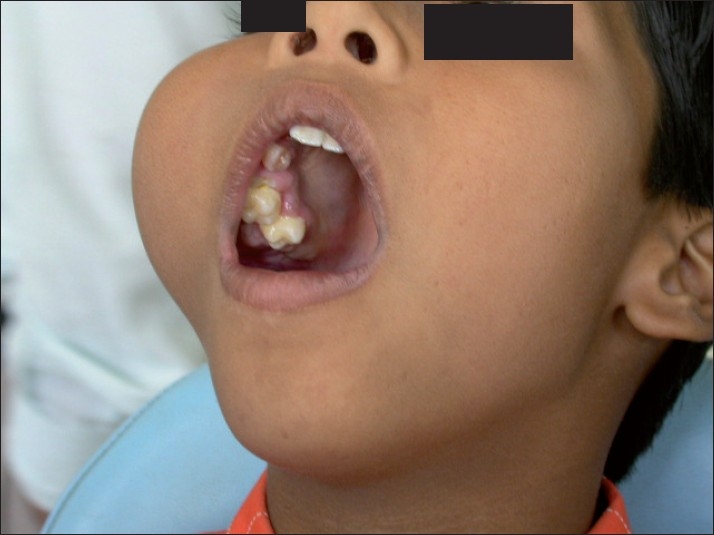
Bony hard swelling extending from the maxillary right lateral incisor to the right maxillary tuberosity

Oral examination revealed a nontender, bony hard swelling extending from the maxillary right lateral incisor to the right maxillary tuberosity, thereby obliterating the right buccal vestibule. The associated teeth showed grade-I mobility. However, the adjacent gingiva and oral mucosa appeared normal [Figure 2].

### Imaging examinations

**Figure 3:**
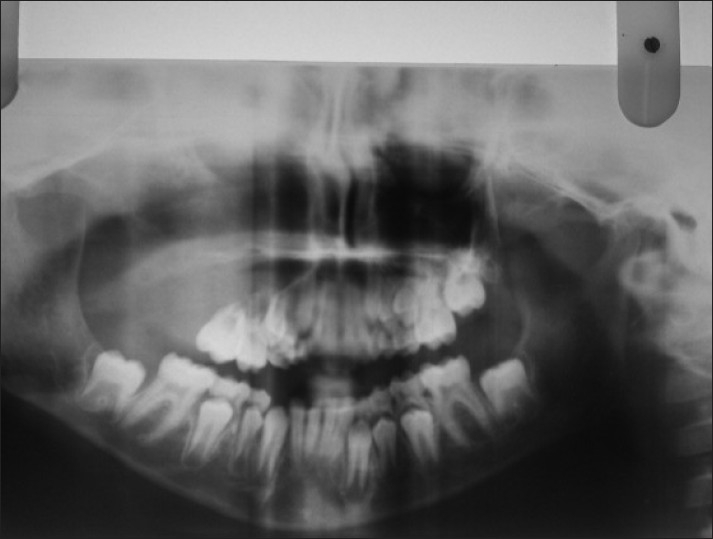
Orthopantamograph showing a unilocular radiolucent lesion extending from 14 to 17

**Figure 4:**
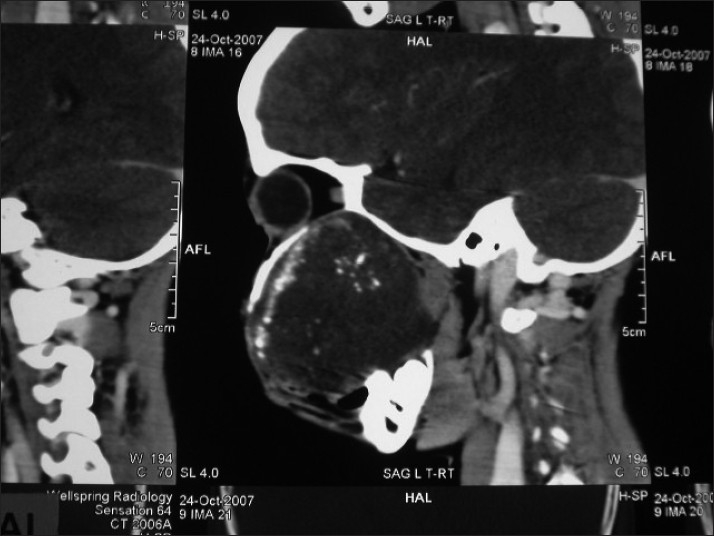
Computed tomographic image showing a single large expansile radiolucent lesion with multiple radioopaque foci

**Figure 5:**
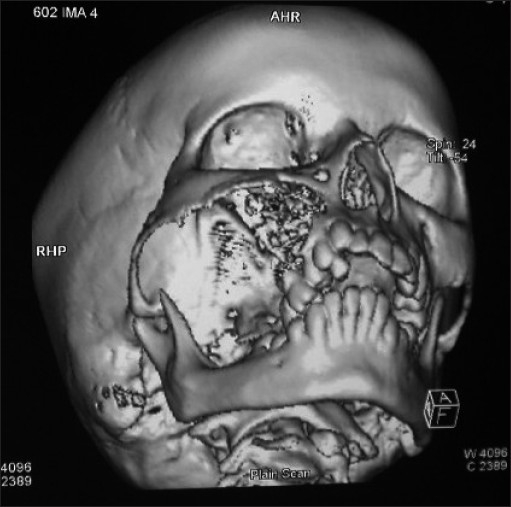
Computed tomographic image showing involvement of the maxillary sinus with the erosion of alveolar bone and medial, lateral and superior walls of the sinus

The orthopantamograph revealed a unilocular radiolucent lesion extending from 14 to 17. Teeth in the affected region showed displacement and root resorption [Figure 3]. Computed tomographic images showed a single large expansile radiolucent lesion with multiple radioopaque foci seen on the right side of craniofacial region, involving the maxillary sinus with erosion of the alveolar bone and medial, lateral and superior walls of the sinus [Figures 4 and 5].

### Cytopathological and histopathological findings

Fine needle aspiration cytology showed few stellate and spindle-shaped cells within the myxoid background.

**Figure 6:**
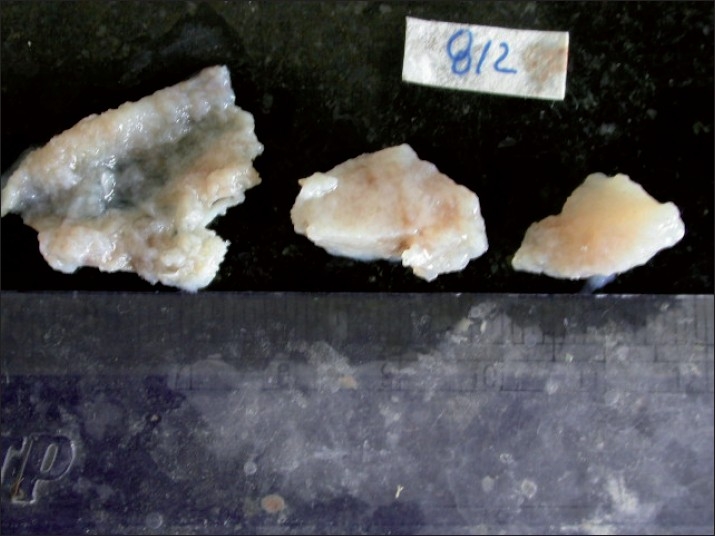
Gross specimen showing a smooth, glistening, gelatinous, lobulated mass

On gross examination, the incisional biopsy specimen appeared as a smooth, glistening, gelatinous, lobulated mass. Its color varied from grayish-white to yellow [Figure 6].

**Figure 7:**
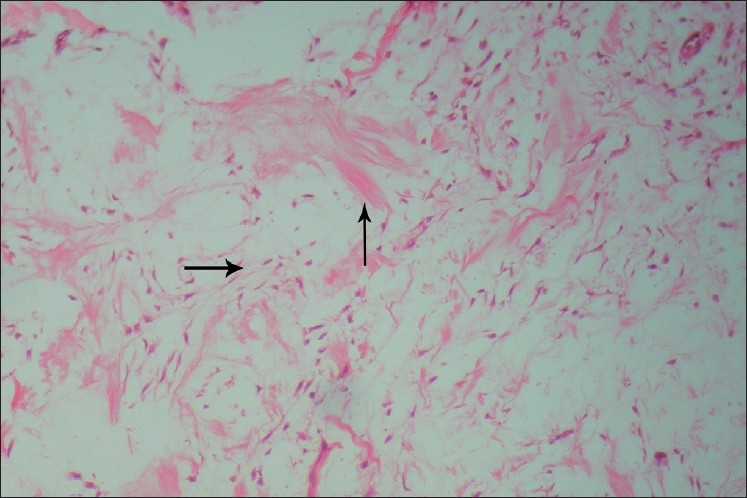
Stellate reticulum-like cells and bundles of collagen fibers in a myxomatous background, H and E, ×100

**Figure 8:**
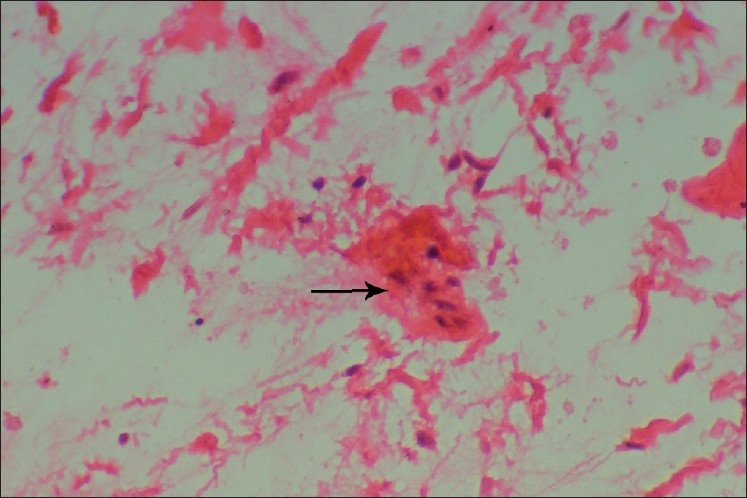
Odontogenic islands within the stroma, H and E, ×400

Histopathological examination of the biopsy specimen revealed the typical features of a myxoma, containing loosely arranged stellate or spindle-shaped cells within a myxoid matrix. Few bony spicules with osteoblastic rimming were also seen at the periphery of the lesion [Figure 7]. At places, the tumor showed bundles of collagen fibers. Islands or nests of odontogenic epithelium were also seen scattered throughout the tumor mass [Figure 8].

### Treatment and prognosis

Under the histopathologic diagnosis of odontogenic fibromyxoma, the patient underwent removal of the tumor with a partial *en bloc* excision of the maxilla. This mode of treatment was selected because of the high recurrence rate of these tumors after conservative treatment. The course has been uneventful for 6 months after surgical removal of the tumor.

## DISCUSSION

Odontogenic myxoma (fibromyxoma) is a benign neoplasm of uncertain histogenesis with a characteristic histologic appearance. It often shows infiltration and behaves in a locally aggressive fashion.[5]

There has been a great deal of controversy regarding the origin of myxomatous tumors. Virchow, in 1863, coined the term myxoma for a group of tumors that had histologic resemblance to the mucinous substance of the umbilical chord.[6] In 1948, Stout redefined the histologic criteria for myxomas as true neoplasms that do not metastasize and exclude the presence of recognizable cellular components of other mesenchymal tissues, especially chondroblasts, lipoblasts and rhabdomyoblasts. Myxoma is a tumor that can be found in heart, skin and subcutaneous tissue and, centrally, in the bone.

Myxomas of the head and neck are rare tumors. Two forms can be identified: (1) facial bone derived, which had been subdivided in the past into true osteogenic myxoma and odontogenic myxoma and (2) “soft tissue”-derived myxoma, derived from the perioral soft tissue, parotid gland, ear and larynx.[7]

Traditionally, the myxoma of the maxilla and mandible has been considered to be a neoplasm of odontogenic origin. Although the evidence is mainly circumstantial, support of an odontogenic origin has been perpetuated by its almost exclusive occurrence in the tooth-bearing areas of the jaws, its common association with an unerupted tooth or a developmentally absent tooth, its frequent occurrence in young individuals, its histologic resemblance to dental mesenchyme, especially the dental papilla and the occasional presence of sparse amounts of odontogenic epithelium.[8]

In a recent immunohistochemical and ultrastructural study, Moshiri *et al*. supported the notion of odontogenic origin of myxomas by suggesting that fibroblasts that compose the tooth germ undergo modification to give rise to odontogenic myxoma.[9]

Slootweg and Wittkampf on the other hand showed that the matrix of myxomas of the jaw is entirely different from the matrix seen in the dental pulp and periodontal ligament. In addition, they also argued that myxomas may also develop in the sinonasal tract and other facial bones that originate from the nonodontogenic mesenchyme. According to them, even the presence of odontogenic epithelium is not necessary to make the diagnosis of myxoma of bone.[10]

Contrary to the findings of Slootweg and Wittkampf, McClure and Dahlin reviewed more than 600 bone tumors of patients at Mayo Clinic and concluded that there were no true myxomas of the bone except for those found in the mandible and maxilla.[11]

Kaffe *et al*. reviewed 164 odontogenic myxomas of the jaw and found that 75% occurred between the second and fourth decades (patient age range, 1–73 years; mean 30 years).[12]

Farman *et al*. differentiated between maxillary and mandibular odontogenic myxomas and suggested that the mean age at the time of diagnosis of maxillary odontogenic myxomas in men was 29.2 years and in women was 35.3 years, while the mandibular odontogenic myxomas in men occur at a mean age of 25.8 years and in women they occur at 29.3 years.[13] Gunhan *et al*.[14] and Regezi *et al*.[7] reported a higher incidence of these tumors in women (64–95%) than in men.[8]

Harder held the view that the odontogenic myxomas only rarely occur before the age of 10 years.[15] However, we present a rare case of odontogenic myxoma occurring in the maxilla of a 7-year-old male patient.

Mandibular myxomas accounted for 66.4%, with 33.6% in the maxilla. Whereas 65.1% of the mandibular cases were located in the molar and premolar areas, 73.8% cases were seen in the same areas of the maxilla.[16] In the present case, the lesion was located in the premolar and molar area of the maxilla.

Most odontogenic myxomas are first noticed as a result of a slowly increasing swelling or asymmetry of the affected jaw. Lesions are generally painless and ulceration of the overlying oral mucosa only occurs when the tumor interferes with dental occlusion. Growth may be rapid and infiltration of neighboring soft tissue structure may occur. Both the buccal and the lingual cortical plates of the mandible may expand occasionally.[17] Kaffe *et Al*. found expansion of the jaws in 74% of the cases. When the maxillary sinus is involved, the odontogenic myxomas often fill the entire antrum. In severe cases, nasal obstruction or exopthalmus may be the leading symptoms.[12] Although exopthalmus was not noticed in the present case, the right lower eyelid and the eyeball were pushed upward. Displacement of teeth has been registered in 9.5% of the cases. Both the features were appreciated in the present case.

On conventional radiographs, myxomas of the jaws often show multilocular radiolucencies representative of “honey comb,” “soap bubble” or “tennis racquet” appearance, which helps in distinguishing this entity from malignant tumors arising centrally within the jaw bones, because the latter usually cause massive bone destruction without compartments formed by bony trabeculations or bony septa.[18] In the present case, the orthopantamograph revealed a single large expansile radiolucent lesion without any trabeculations in the area of bony destruction. However, few radioopacties were seen within the radiolucency.

Computed tomographic images of odontogenic myxomas may show any of the following features:

- Osteolytic expansile lesions with mild enhancement of the solid portion of the mass in the myxoma of the mandible.
- Bony expansion and thinning of cortical plates with strong enhancement of the mass lesion in the anterior maxilla.
- A soft tissue mass with bone destruction and thinning and strands of fine lace-like density representing ossifications in the maxillary sinus.[3]

A similar finding was noticed in the computed tomographic image of the present case. Magnetic resonance imaging (MRI) revealed a well-defined, well-enhanced lesion with homogenous signal intensity on every pulse sequence. The lesion showed intermediate signal intensity on the T1-T2-weighted images.[16] Unfortunately, MRI was not performed in this case.

On gross examination of the specimen, the gelatinous, loose structure of the myxoma was obvious.[19]

Microscopically, the myxoma is made up of loosely arranged spindle-shaped and stellate cells, many of which have long fibrillar processes that tend to intermesh. The loose tissue is not highly cellular, and these cells do not show evidence of significant activity (pleomorphism, prominent nucleoli or mitotic figures). The intercellular substance is mucoid. The tumor is usually interspersed with a variable number of tiny capillaries and occasionally strands of collagen.[20] In case of fibromyxoma, the amount of collagen in the mucoid stroma is more prominent. The fibrils have been shown by silver impregnation to be reticulin. Remnants of odontogenic epithelium have occasionally been noted, sometimes being surrounded by a narrow zone of hyalinization. The myxomatous component of odontogenic myxomas has been compared with the primitive mesenchyme that is found throughout the body. It has also been compared with the dental papilla and the dental follicle.[16]

Farman *et al*. reviewed the histochemical findings in odontogenic myxomas. The ground substance of odontogenic myxomas has been shown to consist of about 80% hyaluronic acid and 20% chondroitin sulfate. Tumor cells appear to be relatively inactive, with low levels of oxidative enzymes. Tumor cells also show slight alkaline phosphatase activity. The myxoid intercellular matrix stains positively with alcian blue, but PAS staining may be negative.[13]

Odontogenic myxoma tumor cells are mesenchymal in origin and express vimentin and muscle-specific actin. Conflicting description of S-100 and GFAP positivity has been reported.[21] The matrix exhibits different proteins, mostly type-I and type IV collagen, fibronectin and proteoglycans.[16]

An extensive study on the ultrastructure of odontogenic myxomas was published by Goldblat in 1976. Two basic types of tumor cells were described, secretory and nonsecretory. The secretory cell type was considered the principal tumor cell and resembled fibroblasts.[16] While generally considered a slow-growing neoplasm, odontogenic myxomas may be infiltrative and aggressive, with high recurrence rates. Because of poor follow-up and lack of reports, a precise analysis of recurrence rates is still missing. Treatment of odontogenic myxomas vary from local excision, curettage or enucleation to radical resection. Recurrence is considered to be directly related to the type of therapy, with conservative surgery resulting in a higher number of recurrences. In the present case, the tumor was completely removed by *en bloc* resection and no recurrence was reported even after 6 months of the surgery.

## CONCLUSION

Myxomas of the head and neck are rare tumors of uncertain histogenesis. Extensive emphasis should be levied on to determine the origin of these locally aggressive myxomas and thereby put to rest various controversies surrounding these lesions.
